# Cheaper is not always worse: strongly protective isolates of a defensive symbiont are less costly to the aphid host

**DOI:** 10.1098/rspb.2014.2333

**Published:** 2015-01-22

**Authors:** Luis Cayetano, Lukas Rothacher, Jean-Christophe Simon, Christoph Vorburger

**Affiliations:** 1 Institute of Integrative Biology, ETH Zürich, Universitätsstrasse 16, 8092 Zürich, Switzerland; 2 EAWAG, Swiss Federal Institute of Aquatic Science and Technology, Überlandstrasse 133, 8600 Dübendorf, Switzerland; 3 INRA, UMR 1349 INRA-Agrocampus Ouest/Université Rennes 1, Institut de Génétique, Environnement et Protection des Plantes (IGEPP), Domaine de la Motte, 35653 Le Rheu Cedex, France

**Keywords:** cost of resistance, defensive symbiosis, *Hamiltonella defensa*, lifespan, parasitoid, trade-off

## Abstract

Defences against parasites are typically associated with costs to the host that contribute to the maintenance of variation in resistance. This also applies to the defence provided by the facultative bacterial endosymbiont *Hamiltonella defensa,* which protects its aphid hosts against parasitoid wasps while imposing life-history costs. To investigate the cost–benefit relationship within protected hosts, we introduced multiple isolates of *H. defensa* to the same genetic backgrounds of black bean aphids, *Aphis fabae*, and we quantified the protection against their parasitoid *Lysiphlebus fabarum* as well as the costs to the host (reduced lifespan and reproduction) in the absence of parasitoids. Surprisingly, we observed the opposite of a trade-off. Strongly protective isolates of *H. defensa* reduced lifespan and lifetime reproduction of unparasitized aphids to a lesser extent than weakly protective isolates. This finding has important implications for the evolution of defensive symbiosis and highlights the need for a better understanding of how strain variation in protective symbionts is maintained.

## 1. Introduction

Life-history costs associated with defences against parasites are well documented [1], and represent a key determinant of host–parasite coevolutionary processes and population dynamics [2–4]. In addition to genotype-by-genotype interactions between hosts and parasites, defence costs can contribute to the maintenance of genetic variation for resistance by negative frequency-dependent selection [5–7]. Resistance to parasites may be costly because it involves the maintenance or deployment of an energetically expensive immune system [8,9], because it trades off against other ecologically relevant traits or because resistance to one parasite may reduce resistance to another [1]. Resistance may also accrue from defences other than the host immune system, such as the possession of heritable endosymbiotic bacteria that protect hosts against parasites [10,11]. This is the case in aphids, which are frequently infected with heritable facultative symbionts that increase their resistance to parasites and pathogens (reviewed in [12,13]). One of these symbionts, *Hamiltonella defensa* [14], provides strong protection against parasitoid wasps [15,16], important natural enemies of aphids. Despite the protection it provides, *H. defensa* does not go to fixation and typically occurs at moderate frequencies in natural aphid populations [17–19]. This may be explained by costs of infection with *H. defensa*, such as lower competitive ability [10] or reduced lifespan and lifetime reproduction [11,20], selecting against symbiont-protected aphids when parasitoids are rare or absent. Different strains of *H. defensa* can differ dramatically in the strength of resistance they provide [16,21], although the level of protection may also depend on the genotype of the attacking parasitoid as a result of genotype-by-genotype interactions between defensive symbionts and parasitoids [16,22,23]. There is also evidence for variation among symbiont strains with respect to life-history costs imposed on the host in the form of reduced lifespan and lifetime reproduction [11]. Heretofore uninvestigated is how these traits are related (but see [24]). Life-history costs can contribute to the evolutionary maintenance of strain diversity in defensive symbionts if they scale with the benefits. This study directly addresses this question to ascertain whether, as per standard expectations for defences against parasites [25], more protective symbionts are also more costly to the host. Surprisingly, we found the opposite, suggesting that the cost–benefit relationship for protective endosymbionts may be fundamentally different to that typically found for other defences.

## 2. Material and methods

### (a) Study system

The black bean aphid, *Aphis fabae*, is a significant pest on broad beans (*Vicia faba*) and beets (*Beta* sp.). It is most abundant in temperate regions of the Northern Hemisphere, where it reproduces by cyclical parthenogenesis. Many clonal generations of live-bearing females during the growth season are followed by a single sexual generation in autumn that produces overwintering eggs. Based on a survey of over 400 individuals collected in Switzerland and eastern France, just over half of the population is infected with *H. defensa* (51% [26]). As originally discovered in pea aphids (*Acyrthosiphon pisum*), which enjoy increased resistance to their parasitoids *Aphidius ervi* and *Aph. eadyi* when harbouring *H. defensa* [15,27], this bacterium also protects black bean aphids against their main parasitoid, *Lysiphlebus fabarum* [16,17]. This resistance requires the presence of toxin-encoding bacteriophages known as *Acyrthosiphon pisum* secondary endosymbionts (APSEs) in the *H. defensa* genome [28,29]. In susceptible aphids, the egg injected by the female parasitoid hatches and develops through four larval instars inside the still growing aphid before it kills and ‘mummifies’ its host by spinning a pupation cocoon inside the aphid's exoskeleton. After metamorphosis, the adult wasp ecloses from this mummy. *Lysiphlebus fabarum* is unique among aphid parasitoids in that most populations are all-female and reproduce by thelytoky [30,31] (i.e. asexual females produce diploid daughters from unfertilized eggs). Diploidy is restored by central fusion automixis [32], which entails that asexual lines rapidly lose heterozygosity in recombining regions of the genome and thus are genetically uniform, even though they are not truly clonal. The possibility to use asexual lines of host and parasitoid makes the *A. fabae*/*L. fabarum* system a powerful model to study genotype-by-genotype interactions between host and parasitoid.

### (b) Experimental lines

We compared the effects of 11 different isolates of *H. defensa* on resistance to parasitoids and aphid life-history traits*.* These isolates were obtained from 11 different field-collected clones of *A. fabae* harbouring natural infections with this symbiont. The aphid clones were founded from single females collected during a Europe-wide sampling effort [33] and maintained since collection in the laboratory on broad bean plants under summer-like conditions that guarantee continued clonal reproduction (18–20°C, 16 h photoperiod). They were chosen to represent a wide range of resistance to *L. fabarum* based on earlier experiments [17,34]. To estimate the symbiont's effects unconfounded by host genetic variation, we introduced *H. defensa* from the 11 donor clones into the same genetic backgrounds represented by two *H. defensa*-free clones of *A. fabae* (nos. 405 and 407) collected during the same sampling campaign. The two recipient clones were uninfected with any known secondary symbionts of aphids [17]. Collection details of the donor and recipient clones are provided in electronic supplementary material, table S1. The recipient clones were experimentally infected by microinjection of haemolymph from each of the donor clones, using the protocol described in [35]. Microinjections took place between 10 and approximately 100 generations prior to the lines' use in the present experiment, resulting in stable, heritable infections that were confirmed by diagnostic PCR repeatedly and again immediately before their use. This procedure resulted in 22 infected aphid sublines, 11 of each clone. Together with two sublines of each clone that remained uninfected with *H. defensa*, they comprised the set of 24 sublines used for the experiments. The uninfected sublines were designated simply as 405 and 407, respectively; the infected sublines were labelled using superscripts denoting symbiont isolates (e.g. 407^H9^ is clone no. 407 infected with *H. defensa* from clone no. 9). To assess molecular variation among *H. defensa* isolates, we sequenced fragments of two bacterial housekeeping genes, acetyl-CoA carboxylase (*accD*) and Murein (*murE*). DNA was extracted from five aphids from each transfected subline of clone 407 using the ‘salting out’ protocol [36]. Fragments were amplified by PCR using *H. defensa*-specific primers and cycling conditions described in [37]. Amplicons were sent to Genoscreen for Sanger sequencing. As parasitoids, we used five different asexual isofemale lines of *L. fabarum* collected from *A. fabae* in the field (electronic supplementary material, table S1). Since their collection, they have been maintained in the laboratory on a standard, *H. defensa*-free clone of *A. fabae* that was not included in the experiment. These lines were chosen to cover a wide range of effectiveness in parasitizing different *A. fabae* clones as judged from earlier experiments [16,22,34] (L.C., L.R., J.-C.S. & C.V. 2009, unpublished data).

### (c) Experimental design and procedures

#### (i) Experiment 1—protection against parasitoids conferred by different isolates of *Hamiltonella defensa*

This experiment quantified the resistance conferred by the different isolates of *H. defensa* against *L. fabarum*. Each of the 24 aphid sublines mentioned above was exposed to each of the five parasitoid lines in a full factorial design with 120 different host subline × parasitoid line combinations. Each combination was replicated three times for a total of 360 replicates. One replicate per combination was processed on each of three consecutive days (three experimental blocks). Similar to previous experiments [17], the assays consisted of exposing groups of aphid nymphs to wasps for a fixed amount of time and determining the proportion of individuals that get mummified (i.e. successfully parasitized). In order to make replicates truly independent and preclude between-line variation accruing from environmental maternal and/or grand-maternal effects carried over from the stock culture, we maintained the 360 aphid colonies for two generations before the start of actual experimental treatments. Fresh seedlings of broad beans grown in 0.07 litre plastic pots were used in each generation, covered by cylindrical plastic cages that had one end covered with gauze. To initiate the test generation, three adults were transferred on new plants to reproduce for 24 h and then removed. The resulting nymphs in each cage were counted 48 h later. Colonies had a mean size of 21.9 ± 6.6 (s.d.). These aphid nymphs (48–72 h old at this stage) were then exposed to a single wasp per cage. Wasps were removed again after 12 h. Nine days after aphids were exposed to wasps, successfully parasitized individuals had turned into mummies and were counted along with the surviving individuals from the original cohort of nymphs (now adults).

Susceptibility to parasitoids was quantified in two ways: as the proportion of all aphids exposed to parasitoids that became mummified, and as the proportion of mummies among all individuals still present on the plant on the day of counting (ignoring individuals that died in the 9 days between exposure to wasps and counting, which were therefore unaccounted for). However, the results were qualitatively the same, hence we report only the analysis of the proportion mummified of all individuals exposed to wasps. Owing to handling errors, three replicates had to be excluded from the analysis. Strong overdispersion precluded the use of a generalized linear model with binomial errors to analyse these proportion data, thus we resorted to a linear model on arcsine-square-root-transformed proportions. This transformation improved, but did not fully achieve homogeneity of variances (*F*_71,287_ = 1.688, *p* = 0.002) and normality of residuals (K–S test, *p* = 0.001), presumably owing to many values of zero in lines with complete or near-complete resistance. This necessitates careful interpretation of marginal *p*-values, even though linear models are fairly robust to violations of parametric assumptions [38]. We tested for the effects of block, host genetic background (‘aphid genotype’), parasitoid line, aphid subline (or symbiont haplotype—see Results) and their interactions. The model was run once using all aphid sublines and once restricted to symbiont-infected sublines, because only in the latter case are interactions involving aphid subline strictly interpretable as interactions mediated by symbiont strain. All statistical analyses were performed using SPSS/PASW 18 (IBM, Armonk, NY).

#### (ii) Experiment 2—life-history costs induced by different isolates of *Hamiltonella defensa*

Before exposure to parasitoids, one aphid nymph was removed from each of eight colonies of all 24 aphid sublines (three from the first two experimental blocks, two from the third block), yielding 192 nymphs in total. These nymphs were transferred singly to new plants and used to measure three life-history traits: weight at adulthood, lifespan and total lifetime reproduction. When aphids reached adulthood, their weight was measured on an electronic scale (model MX5, Mettler Toledo, Greifensee, Switzerland) and recorded to the nearest microgram. They were then returned to their plants to reproduce, with a check of survivorship being conducted every 2 days. Every 4 days, the adults were transferred to fresh plants and all their offspring counted on the old plant. The date of death for each adult was recorded to calculate lifespan, and the sum of all offspring produced was our measure of total lifetime reproduction.

Life-history traits were also analysed with linear models, testing for the effects of block, aphid genotype, aphid subline and their interaction. To detect possible relationships between protection and costs to the host conferred by the different *H. defensa* isolates, we calculated Pearson correlations between life-history traits and rates of parasitism averaged across aphid genotypes and parasitoid lines.

#### (iii) Experiment 3—symbiont densities

To check whether bacterial density had any effects on life-history traits and the level of protection against parasitism, we estimated *H. defensa* densities relative to aphid size using TaqMan real-time quantitative PCR on an ABI 7500 fast real-time PCR system (Applied Biosystems, Foster City, CA). DNA was extracted from five replicate adults (9 days old) of each infected aphid subline using the ‘salting out’ method [36] and resuspended in 80 μl of 1× TE buffer. Each aphid was reared on a separate plant to ensure independence of biological replicates. The copy number of *H. defensa*'s *dnaK* gene served as an estimate of symbiont density and the copy number of *A. fabae*'s *EF1α* gene as an index of host cell number. The primer and probe sets used are provided in reference [16]. The 25 μl-volume qPCRs were run in triplicate (technical replicates) with 5 μl of template DNA. Gene copy number was estimated based on a standard curve produced with serial dilutions of a synthetic standard provided by Microsynth AG (Balgach, Switzerland). One sample of 407^H85^ was excluded from the analysis, because the values of its three technical replicates diverged greatly from one another. For another sample of 407^H85^, one of the technical replicates was not used to calculate the sample mean because its value was clearly an extreme outlier resulting from a handling error. Linear models were used to test for the effects of aphid genotype, symbiont isolate (or symbiont haplotype—see Results) and their interactions on relative symbiont density (symbiont gene copy number divided by aphid gene copy number).

## 3. Results

### (a) Endosymbiont sequence types

Comparison of two endosymbiont gene fragments (see Methods) revealed three distinct haplotypes among our isolates: ‘haplotype 1’ comprising isolates H76 and H101, ‘haplotype 2’ comprising AF6, H9, H28, H30, H323, H343 and H402, and ‘haplotype 3’ comprising H15 and H85. Using the concatenated gene fragments, there was a sequence divergence of 0.99% between haplotypes 1 and 2, of 1.32% between 1 and 3, and of 1.16% between 2 and 3. The *accD* and *murE* partial sequences are deposited in GenBank (accession nos. KP071733–KP071738), and the alignments are provided in the electronic supplementary material, figure S1.

### (b) Protection provided by different isolates of *Hamiltonella defensa*

**Figure 1.**
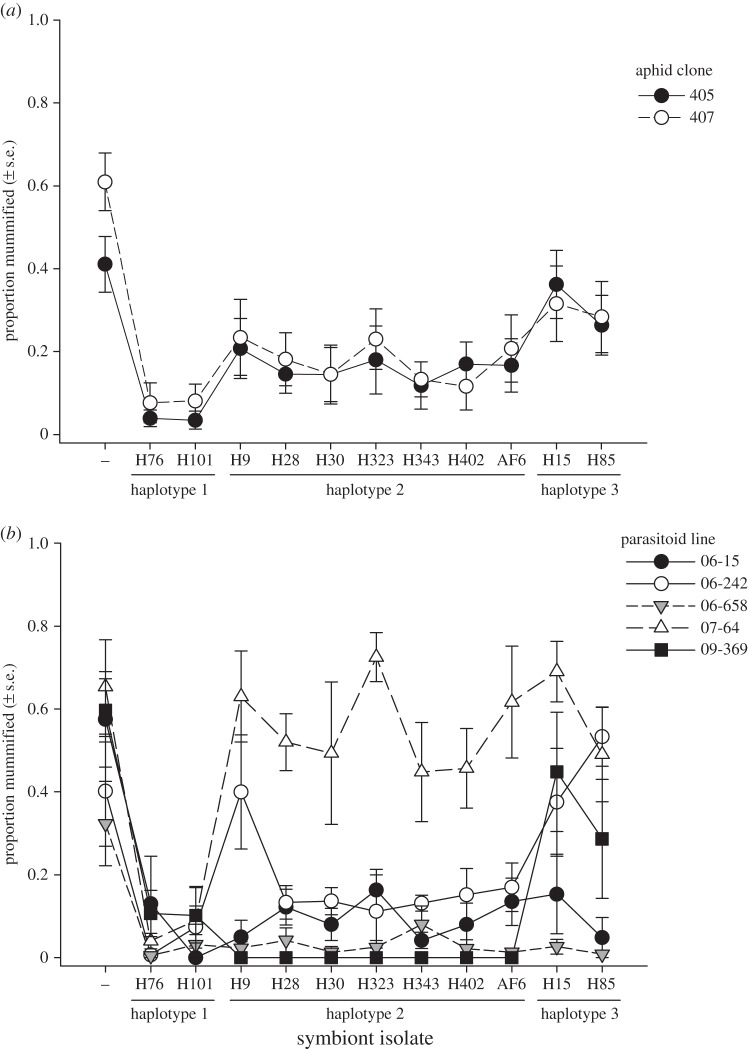
Protection of black bean aphids (*Aphis fabae*) by 11 isolates of the bacterial endosymbiont *Hamiltonella defensa* depends on the genotype of the parasitoid *Lysiphlebus fabarum*, but not on the host's genotype. Symbols depict the rate of parasitism expressed as the proportion of individuals mummified among all aphids exposed to parasitoids (*a*) averaged across all five parasitoid lines and (*b*) averaged across both aphid clones.

As expected, *Hamiltonella*-infected sublines were on average less susceptible to parasitism than uninfected sublines (*F*_1,345_ = 62.935, *p* < 0.001; figure 1*a*). Parasitoid lines differed strongly in infectivity (table 1 and figure 1*b*), which was especially apparent with respect to lines 07-64 and 06-658, having consistently high and low parasitism success, respectively, across most aphid sublines. The protection provided by the different *H. defensa* isolates depended on the parasitoid genotype, as indicated by the highly significant interaction between parasitoid line and aphid subline/symbiont isolate (table 1*a* and figure 1*b*). Nevertheless, the main effect of aphid subline/symbiont isolate was also highly significant (table 1*a*), indicating that some symbionts are more protective than others when averaged across parasitoid lines (table 1*a* and figure 1*a*).

**Table 1.** General linear model results for the proportion of aphids parasitized (arcsine-square-root-transformed). Separate analyses are shown using (*a*) individual aphid sublines and (*b*) sublines pooled together based upon the haplotype group to which their symbionts belong. The analyses were performed once for all aphid sublines and once for only those sublines infected with *H. defensa*, since only in the latter case does the parasitoid line × aphid subline interaction properly reflect genotype × genotype interactions between parasitoids and symbionts/haplotypes.

source	all aphid sublines				*Hamiltonella*-infected sublines only			
	d.f.	MS	*F*	*p*	d.f.	MS	*F*	*p*
(*a*) individual aphid sublines								
block	2	0.502	7.462	0.001	2	0.499	8.012	<0.001
aphid genotype	1	0.131	1.952	0.164	1	0.046	0.734	0.393
aphid subline	11	0.871	12.939	<0.001	10	0.376	6.045	<0.001
parasitoid line	4	4.070	60.466	<0.001	4	4.197	67.453	<0.001
aphid genotype × a. subline	11	0.050	0.741	0.699	10	0.033	0.533	0.866
aphid genotype × parasitoid line	4	0.112	1.670	0.158	4	0.142	2.281	0.062
A. subline × parasitoid line	44	0.224	3.324	<0.001	40	0.213	3.417	<0.001
aphid gen. × a. subline × parasitoid l.	44	0.061	0.909	0.637	40	0.062	0.996	0.485
residual	235	0.067	—	—	215	0.062	—	—
(*b*) symbiont haplotype groups								
block	2	0.492	7.565	0.001	2	0.490	8.014	<0.001
aphid genotype	1	0.208	3.196	0.075	1	0.012	0.200	0.655
symbiont haplotype	3	3.075	47.296	<0.001	2	1.698	27.769	<0.001
parasitoid line	4	1.501	23.081	<0.001	4	2.076	33.955	<0.001
aphid genotype × symb. haplotype	3	0.099	1.522	0.209	2	0.040	0.660	0.518
aphid genotype × parasitoid line	4	0.060	0.926	0.449	4	0.147	2.400	0.050
parasitoid line × symb. haplotype	12	0.622	9.564	<0.001	8	0.766	12.531	<0.001
aphid gen. × parasitoid l. × symb. h.	12	0.092	1.419	0.156	8	0.112	1.826	0.072
residual	315	0.065	—	—	295	0.061	—	—

Symbiont isolates with the same haplotype behaved similarly (figure 1). Pooling symbionts into their respective haplotypes in the analysis yielded qualitatively similar results, though the interaction between aphid genotype and parasitoid line became marginally significant when considering only *Hamiltonella*-infected sublines (table 1*b*).

The two aphid genotypes did not differ significantly in their susceptibility to parasitoids (table 1 and figure 1*a*), nor was there a significant interaction between aphid genotype and subline/symbiont isolate (table 1*a*). These results indicate that the symbionts were the main determinants of susceptibility to parasitoids and that they had similar effects in the two aphid genotypes. There was also a significant block effect on rates of parasitism (table 1).

In many replicates, some aphids that were not mummified by parasitoids nevertheless died before parasitism was quantified. This was particularly true for the most short-lived sublines harbouring isolate H85, whose high intrinsic mortality prevented many individuals from reaching the age at which mummies were counted (12 days), and which therefore could not be considered. For all other isolates of *H. defensa*, host mortality not due to parasitism was negatively related to the susceptibility to parasitoids (i.e. the proportion of individuals mummified; *r* = −0.788, *p* = 0.007).

### (c) Life-history trait variation induced by different isolates of *Hamiltonella defensa*

**Figure 2.**
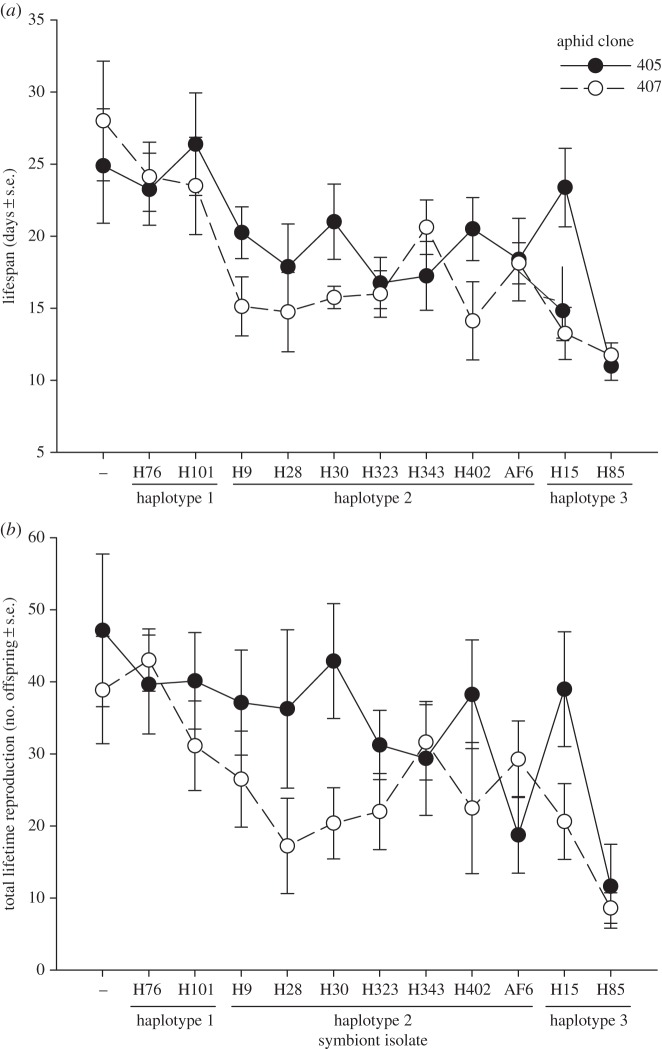
Variation among 11 isolates of the bacterial endosymbiont *Hamiltonella defensa* in the life-history costs imposed on their host, *Aphis fabae*. (*a*) Mean lifespan and (*b*) mean lifetime reproduction of the uninfected and infected sublines from both aphid clones.

Aphid weight at adulthood was not affected significantly by aphid genotype, aphid subline or their interaction (electronic supplementary material, table S2). On the other hand, there was considerable variation in the lifespan of aphids (figure 2*a*). Aphids infected with *H. defensa* had shorter lifespans on average than uninfected aphids (*F*_1,188_ = 16.353, *p* < 0.001). Among aphids harbouring *H. defensa*, there was also significant variation among sublines (*F*_10,152_ = 4.995, *p* < 0.001). Those infected with isolate H85 were the most short-lived, having a mean lifespan of only 11.4 days averaged across both aphid genotypes, whereas aphids infected with isolates H76 and H101 were the most long-lived, with mean lifespans of 23.7 and 24.9 days, respectively, which was very similar to those of uninfected aphids (26.4 days). There was also a significant effect of aphid genotype on lifespan (*F*_1,166_ = 4.443, *p* = 0.037), but no significant aphid genotype × subline interaction, independent of whether all aphid sublines were considered (*F*_11,166_ = 1.337, *p* = 0.208) or only those harbouring *H. defensa* (*F*_10,152_ = 1.461, *p* = 0.159). Clone 405 was more long-lived than 407 overall, even though uninfected aphids of clone 407 had slightly longer lifespans (figure 2*a*). It thus appears that the presence of *H. defensa* has a stronger negative effect on clone 407 than on clone 405, which is consistent with an earlier study [11].

The negative effect on lifespan imposed by harbouring *H. defensa* translated into similar patterns for lifetime reproduction (figure 2*b*). Aphids harbouring *H. defensa* produced fewer offspring on average than aphids without the symbiont (*F*_1,188_ = 7.004, *p* = 0.009), and the magnitude of the decrease depended on the subline, which had a significant main effect on lifetime reproduction (*F*_10,152_ = 2.942, *p* = 0.002; only infected aphids considered), but did not show an interaction with aphid genotype (*F*_10,152_ = 1.344, *p* = 0.212). Among the infected aphids, the most short-lived sublines possessing isolate H85 also produced the fewest offspring (10.1 on average), and the most long-lived sublines possessing H76 and H101 produced the most (41.3 and 35.6), only slightly fewer than the uninfected sublines (43.0). The two aphid genotypes also differed significantly (*F*_1,166_ = 9.281, *p* = 0.003), with clone 405 producing more offspring on average than clone 407 (figure 2*b*).

Symbiont isolates with the same haplotype behaved similarly with respect to their effects on aphid lifespan and lifetime reproduction, albeit not to the same extent as for protection against parasitoids (figure 2). Accordingly, symbiont haplotype had a significant main effect on both life-history traits (lifespan: *F*_2,168_ = 18.079, *p* < 0.001; lifetime reproduction: *F*_2,168_ = 7.868, *p* = 0.001; only infected aphids considered). The complete analyses for lifespan and lifetime reproduction (equivalent to table 1) are provided in the electronic supplementary material, tables S3 and S4.

### (d) Relationship between protection and life-history traits

**Figure 3.**
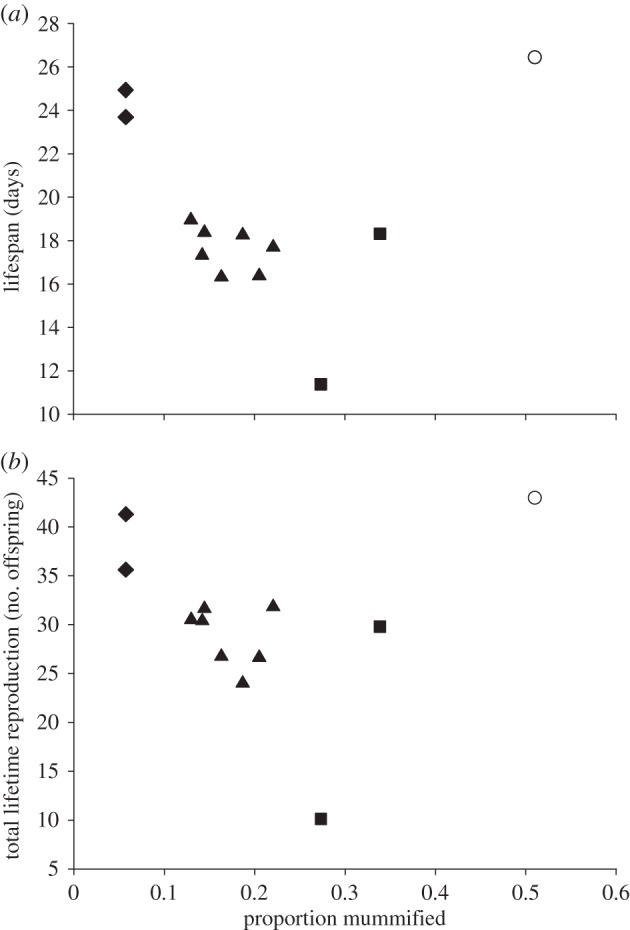
More protective isolates of *Hamiltonella defensa* are less detrimental for the aphid host in the absence of parasitoids. Scatter plots show the relationship between (*a*) lifespan and (*b*) total lifetime reproduction in the absence of parasitoids and the susceptibility to parasitoids expressed as the proportion of aphids mummified, averaged across all parasitoid lines and both aphid genotypes. Different symbols denote different symbiont haplotypes (diamonds, haplotype 1; triangles, haplotype 2; squares, haplotype 3). Open circles represent uninfected aphids for reference.

When averaged over aphid clones and parasitoid lines, the rate of parasitism experienced by aphid sublines harbouring different isolates of *H. defensa* exhibited a significant negative correlation with lifespan (*r* = −0.705, *p* = 0.015), as well as with total lifetime reproduction (*r* = −0.625, *p* = 0.040). These correlations are illustrated in figure 3, which also shows that isolates belonging to the same haplotype cluster closely together, except for the two isolates belonging to haplotype 3. Haplotype 1 comprised two isolates (H76 and H101) that provide strong protection against parasitoids, and have little effect on host lifespan and reproduction. Haplotype 2 comprised seven isolates that provide medium protection, and cause moderate reductions of lifespan and reproduction. Haplotype 3 comprised two isolates (H15 and H85) that provide relatively weak protection, but only H85 depresses host lifespan and reproduction very strongly. H15 has more moderate effects, comparable to those of isolates from haplotype 2. Overall, this result indicates that in *H. defensa*-infected aphids, increased protection by symbionts is not balanced by negative fitness effects in the absence of parasitoids. More protective isolates were less rather than more costly to the host.

Neither the life-history traits nor the susceptibility to parasitoids (averaged over parasitoid genotypes) of aphid sublines harbouring *H. defensa* were related to the number of generations since the experimental introduction of the symbiont by microinjection (lifespan: *r* = 0.097, *p* = 0.668; lifetime reproduction: *r* = 0.194, *p* = 0.386; susceptibility to parasitoids: *r* = −0.158, *p* = 0.481).

### (e) Symbiont densities

**Figure 4.**
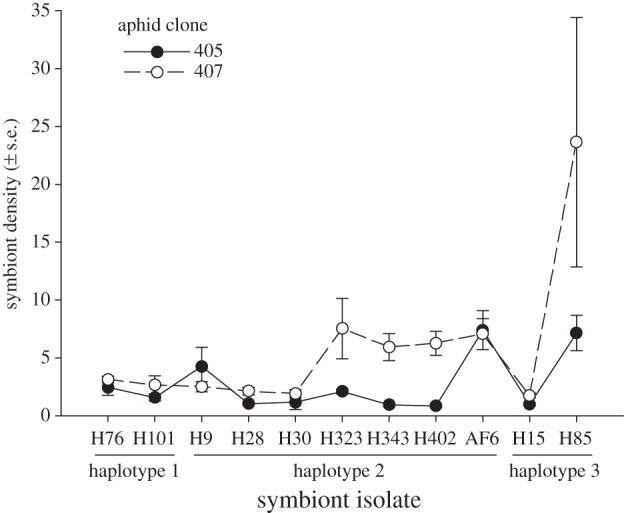
Relative symbiont densities in adult aphids (symbiont gene copy numbers divided by host gene copy numbers) estimated by quantitative PCR for 11 isolates of *Hamiltonella defensa* in the two different clones of *Aphis fabae* used.

Symbiont densities, expressed as the ratio of *H. defensa dnaK* gene copy numbers to *A. fabae EF1α* gene copy numbers, are depicted in figure 4. Aphid genotype had a significant effect on symbiont densities (*F*_1,87_ = 12.967, *p* = 0.001): most isolates of *H. defensa* reached higher densities in clone 407. There was also significant variation among symbiont isolates (*F*_10,87_ = 7.247, *p* < 0.001). The density of H85, the most harmful isolate in this study, was relatively high, especially in aphid genotype 407, where it also showed high variation among individuals (figure 4). That the densities reached by the different isolates of *H. defensa* depended on the aphid genotype was reflected in a significant aphid genotype × isolate interaction (*F*_10,87_ = 2.808, *p* = 0.005). When averaged over aphid genotypes, the symbiont isolates' densities correlated negatively with their host's lifespan (*r* = −0.642, *p* = 0.033) and lifetime reproduction (*r* = −0.841, *p* = 0.001), but this correlation was largely driven by H85. Without this isolate, the correlations became weak and non-significant (*r* = −0.462, *p* = 0.179 for lifespan; *r* = −0.207, *p* = 0.567 for lifetime reproduction). There was no significant correlation between symbiont density and the sublines' susceptibility to parasitism by *L. fabarum* (*r* = 0.354, *p* = 0.285).

## 4. Discussion

Our experiment confirmed that in black bean aphids, the strength of protection provided by different isolates of the facultative symbiont *H. defensa* depends on the genotype of the attacking parasitoid. Genotype-by-genotype interactions on the outcome of infection are a common observation in host–parasite systems [39,40], but notably, in the present case, they are driven by a genetic interaction between the parasitoid and the host's heritable endosymbiont rather than the host itself [16,22,34]. Such genetic specificity has important implications for host–parasite coevolution, because it may lead to rapid dynamics driven by negative frequency-dependent selection [6,41]. Despite these interactions, there was also a highly significant main effect of *H. defensa* isolate, indicating that some strains of the symbiont are consistently more protective than others when averaged over parasitoid genotypes. Variation in the strength of protection among strains of *H. defensa* is also observed in pea aphids [21], where it could be related to the presence of different variants of APSE (APSE-1–3) within the genomes of different symbiont strains [28,29]. Our most surprising result was that the strength of protection was negatively related to the costs imposed on the host, such that more strongly protective isolates of *H. defensa* were less costly to the host. That is, no trade-off between resistance and lifespan or lifetime reproduction in the absence of parasitoids was observed. This was especially interesting given that a trade-off is clearly evident with regard to the presence of *H. defensa per se*: black bean aphids infected with *H. defensa* are more resistant to parasitoids, but suffer a reduced lifespan [11,13] (see also figure 3 in this paper). It is possible that by using experimentally infected lines, the costs of possessing *H. defensa* are overestimated somewhat compared with the natural situation, because such new host–symbiont associations have not been tested by natural selection in the field. On the other hand, costs have also been observed when naturally infected aphids have been cured of *H. defensa* [42].

We do not currently have a mechanistic explanation for the unexpected relationship between protection and costs to the host conferred by these symbionts. A possible clue comes from a recent study on pea aphids [24], showing that the spontaneous loss of APSE-3 from *H. defensa*'s genome was associated with a loss of protection and a dramatic increase in symbiont abundance, which in turn had a strong negative effect on aphid fitness. The bacteriophage may thus also play a role in controlling symbiont population growth, and phage loss could lead to an association of weak protection with high costs to the host. However, this is unlikely to fully explain our results. Even though the most detrimental isolate H85 showed elevated densities at least in one aphid clone, there was no such relationship for the other isolates. Phage loss is also unlikely, because all of our isolates did provide some level of protection, although the presence of APSE has only been tested and confirmed for two isolates (H323 and H402; P. Lukasik 1 June 2014, personal communication). Alternatively, different symbiont isolates may produce different toxins that vary in their negative side effects on the host itself.

Whatever its mechanistic basis, the observed correlation creates an evolutionary puzzle. How are symbiont strains maintained in the population if they are very costly to the host but provide only limited benefits in terms of protection (e.g. H85)? One possibility is that strong protection is balanced by induced rather than by constitutive costs of defence [43] (i.e. costs that are incurred only when a defence is actually deployed). Induced costs may arise if symbionts are able to increase their density or the release of toxins upon parasitoid attack on the host [44], and if this increase also has detrimental effects on the host. One observation from our experiment was indeed suggestive of such an effect, namely that host mortality not owing to parasitism was negatively related to the susceptibility to parasitoids (i.e. the proportion of individuals mummified). This suggests that aphids experience some mortality upon exposure to parasitoids even when they are not successfully parasitized, and that this mortality is higher in aphids possessing more protective isolates, which would curtail the benefits of symbiont-conferred resistance. However, we cannot exclude that this correlation simply reflects a density effect. The fewer individuals are parasitized, the more healthy individuals live and eventually start reproducing on the plants, and this increased ‘density stress’ may in itself explain the higher mortality observed in colonies with few mummies. Furthermore, a direct experimental test (although including only a single symbiont strain) did not provide any evidence for induced costs of resistance conferred by *H. defensa* [45].

Seemingly maladaptive strains of *H. defensa* providing only limited protection at a high cost to the host may also be maintained by selection if they affect other ecologically relevant traits that were not considered here. We are only beginning to appreciate the multitude of effects exerted by bacterial symbionts on insects in general [46], and on aphids in particular [12]. Some symbionts, including *H. defensa*, are known to assist aphids in coping with thermal stress [47,48]; other symbionts provide protection against entomopathogenic fungi [49,50]. There is also evidence that secondary symbionts can mediate the interactions of aphids with their host plants, affecting performance on particular host plants and potentially influencing host range [18,37,51]. Bacterial endosymbionts may also affect behavioural defences against natural enemies [52,53]. Thus, rather than by a physiological cost affecting host life-history traits, protection may be balanced by ecological costs associated with harbouring *H. defensa*; that is, by maladaptive effects on other ecologically relevant traits, such as reduced defence against natural enemies or restricted host plant range. It will clearly be important that future research investigates multiple traits affected by defensive symbionts and analyses their covariation.

Finally, *H. defensa* strains that are very costly to the host may enjoy a higher propensity for horizontal transmission. It appears that the physiological cost to the host may be explained at least in part by high symbiont titres [24,54], which may simultaneously facilitate horizontal transmission. The main transmission route of *H. defensa* is maternal inheritance, but horizontal transmission has been demonstrated as well, either via males during sexual reproduction [55] or via parasitoid vectors that can transmit bacteria between individuals on their contaminated ovipositors [56]. Both routes are likely to be more effective with high symbiont densities in the donors. High rates of horizontal transmission, just as in a pathogen, would spare the requirement of a net benefit to the host for persistence in the population. It appears unlikely that sufficiently high rates of horizontal transmission could be achieved by any strain of *H. defensa*, but the necessary measurements are yet to be made.

To conclude, we investigated the covariation between protection and life-history costs to the host effected by multiple isolates of *H. defensa*. This revealed the highly unexpected association of weak protection against parasitoids with high costs to the host, and vice versa, opposite to what is typically found in host–parasite systems [1]. If generalizable to other systems in which defences are provided by protective symbionts, such defences do not fit into a simple trade-off scheme that underlies much of the theory on the evolution of resistance. This highlights our still limited understanding of the factors that maintain strain variation in endosymbionts [19], as well as the coexistence of protected and unprotected hosts. Improving this understanding will require a more comprehensive assessment of multiple traits affected by symbionts and measuring their joint effects on fitness in the field rather than the laboratory—a challenging but necessary endeavour.

## Data accessibility

Data presented in this paper are available at http://dx.doi.org/10.5061/dryad.r5954.
